# A modified subclassification to evaluate the survival of patients with N3 gastric cancer: an international database study

**DOI:** 10.1186/s12885-018-5187-7

**Published:** 2019-01-07

**Authors:** Man-Qiang Lin, Jia-Bin Wang, Chao-Hui Zheng, Ping Li, Jian-Wei Xie, Jian-Xian Lin, Jun Lu, Qi-Yue Chen, Long-Long Cao, Mi Lin, Qing-Liang He, Chang-Ming Huang

**Affiliations:** 1 Department of Gastric Surgery, Fujian Medical University Union Hospital, No. 29 Xinquan Road, Fuzhou, 350001 Fujian Province China; 2 Department of General Surgery, Fujian Medical University Union Hospital, Fuzhou, China; 3 Key Laboratory of Ministry of Education of Gastrointestinal Cancer, Fujian Medical University, Fuzhou, China; 4 Fujian Key Laboratory of Tumor Microbiology, Fujian Medical University, Fuzhou, China; 5 Department of Gastrointestinal Surgery, the First Affiliated Hospital of Fujian Medical University, No. 20 Chazhong Road, Fuzhou, 350005 Fujian Province China

**Keywords:** Gastric cancer, N3 classification, Cutoff, Number of metastatic lymph nodes, Prognosis

## Abstract

**Background:**

The eighth TNM classification for gastric cancer categorizes N3 as N3a and N3b in the final pathologic stage. The cutoff for N3a/N3b is defined as 15 metastatic lymph nodes, but the rationale for this cutoff remains unclear. This study aimed to determine the optimal N3a/N3b cutoff and evaluate its prognostic significance.

**Methods:**

An international database was constructed by combining data from patients with N3 gastric cancer and complete five-year follow-up data from the Surveillance, Epidemiology, and End Results program database (*n* = 1833) and the Fujian Medical University Union Hospital database (*n* = 920) (total *n* = 2753). A log-rank test was performed to determine the optimal N3a/N3b cutoff, and its prognostic significance was confirmed in a two-step multivariate analysis and compared to that of the eighth TNM.

**Results:**

A cut-point analysis performed at each metastatic lymph node number identified the greatest survival difference between N3a and N3b at 13 metastatic lymph nodes (χ^2^ = 157.671, *P* = 3.65 × 10^− 36^). In patients with 14–15 metastatic lymph nodes, prognoses were significantly worse than those in patients with 7–13 metastatic lymph nodes (*P* < 0.001) but similar to those in patients with > 15 metastatic lymph nodes (*P* = 0.078). Therefore, patients with 14–15 metastatic lymph nodes were incorporated into a modified N3b classification. In the two-step multivariate analysis, the eighth N3 classification fell out of the model, while the modified N3 classification remained intact (HR 1.51, *P* < 0.001). Further analyses demonstrated that the modified TNM classification had superior homogeneity, discriminatory ability, and gradient monotonicity compared to the eighth TNM classification.

**Conclusions:**

For improved prognostic stratification, we recommend adjusting the cutoff for subclassification of N3 gastric cancer to 13 metastatic lymph nodes.

**Electronic supplementary material:**

The online version of this article (10.1186/s12885-018-5187-7) contains supplementary material, which is available to authorized users.

## Background

Two major classification systems are used for gastric cancer staging. These include the Japanese Classification of Gastric Cancer (JCGC) and the Union for International Cancer Control/American Joint Committee on Cancer (UICC/AJCC) TNM classification system. In previous decades, the ability to accurately stage gastric cancer has continuously improved, and N3 staging has accordingly undergone several revisions. According to the number of metastatic lymph nodes (MLNs), the fifth UICC/AJCC TNM classification defined N3 as > 15 MLNs [1]. However, the early JCGC defined N3 with metastases to Group 3 lymph nodes (LNs) according to the location of the MLNs relative to that of the primary tumor [2, 3]. In 2010, the 14th JCGC was unified with the seventh UICC/AJCC classification system, in which the definition of N3 was modified to > 7 MLNs, and N3 was divided into N3a (7–15 MLNs) and N3b (> 15 MLNs). However, the definitions of the N3 subclasses (N3a and N3b) were not different with regard for the final pathological stage [4, 5].

The seventh UICC/AJCC classification for LN metastases has been described as reliable for predicting prognoses in gastric cancer in many studies. However, the N3 classification provided in this edition is controversial [6, 7]. Sano et al. found that the prognoses of patients with stage N3a and N3b from 15 different countries were distinct [8]. Hence, the eighth TNM classification detailed N3 as N3a and N3b in the final pathological stage of the disease [9].

The cutoff used to distinguish N3a/N3b in the current TNM classification was derived from a German retrospective study that was performed 20 years ago [10]. The study sample was ethnically monotonous and small; therefore, the rationale for adopting 15 MLNs as the cutoff for the two groups remains unclear. Determining an accurate and reasonable N stage is important when planning a treatment strategy, determining a prognosis, evaluating the results of treatment and exchanging information [11]. This study was designed to determine the optimal cutoff for distinguishing N3a/N3b and evaluate its prognostic significance using a newly created combined international dataset.

## Methods

### Patients

An international gastric cancer dataset including Eastern and Western populations was established by combining the Surveillance, Epidemiology, and End Results (SEER) database (http://seer.cancer.gov/) with the Fujian Medical University Union Hospital (FMUUH) database. This study was a retrospective analysis of 1833 patients (SEER) plus 920 patients (FMUUH) with N3 gastric cancer who underwent a gastrectomy between January 1988 and December 2008 and between January 1995 and December 2011, respectively.

The following inclusion criteria were applied [1]: the primary tumor localization was the stomach (SEER: C16.1-C16.9) [2]; the patient was ≥18 years old [3]; histology confirmed an adenocarcinoma (SEER: Type ICD-O-3: 8140, 8142–8145, 8210, 8211, 8255, 8260–8263, 8310, 8323, 8480, 8481, and 8490) [4]; the patient underwent a gastrectomy (SEER: rx sum-surg prim site (1998+): 30–80 and site-specific surgery (1973–1997): 20–78) [5]; > 15 LNs were retrieved [6]; the lesions were staged pT1-4N3M0. The following exclusion criteria were used to rule out patients [1]: histology identified a tumor type other than adenocarcinoma [2], survival < 1 month [3], multiple primary tumors [4], remnant gastric cancer [5], distant metastases were apparent (M1), and [6] incomplete datasets. The stepwise process used to extract data is shown in Additional file 1: Figure S1.

The clinicopathological features obtained for this study included age, gender, ethnicity, histology, tumor size, tumor-node-metastasis (TNM) stage, type of surgery, the number of LNs examined, and the number of MLNs. The patients were classified into age groups of < 65 and ≥ 65 years old based on the WHO definition of “elderly” [12]. Ethnicity was classified as White, Asian, Black, Hispanic and Native American. The tumors were divided by size into tumors that were ≤ 60 mm and > 60 mm in diameter. The type of surgery included proximal/distal gastrectomy and total gastrectomy. Tumor staging was determined based on the eighth edition of the UICC/AJCC TNM classification [9].

Patients in FMUUH were followed up every three months for two years following surgery and then every six months for the next 3–5 years. The majority of patients routinely underwent laboratory tests, chest radiography, abdominopelvic ultrasonography or computed tomography, and annual gastroscopy. Overall survival refers to the period from the day of the operation to the date of death or last follow-up (SEER: December 2013 and FMUUH: December 2016). All survivors were followed up for more than five years.

### Statistical analysis

SPSS 18.0 (SPSS Inc., Chicago, IL) and STATA12.0 statistical software were used to analyze the data. To compare the clinicopathological characteristics between patients in the SEER and FMUUH databases, the χ^2^ test was performed to analyze categorical variables, while unpaired continuous variables were analyzed using the Mann-Whitney U test. To determine the best cutoff for distinguishing N3a/N3b, we evaluated the ability of prognostic stratification at each MLN count value using the magnitude of the log-rank test χ^2^ statistic [13]. The cutoff that appeared to provide the greatest actuarial survival difference between the resulting subgroups was verified by X-tile software [14]. Survival curves were constructed according to the Kaplan-Meier method, and a log-rank test was used to determine whether significant differences were present among survival curves. We used a two-step multivariate analysis to evaluate the validity of the modified N3 classification (mN3) [15]. In the first step, the prognostic factors identified in the univariate analysis were incorporated into the multivariate analysis with the eighth N3 staging criteria but excluding the mN3 criteria; in the second step, the eighth N3 and mN3 staging criteria were simultaneously included in the multivariate analysis. Finally, to compare the prognostic performance of the eighth TNM and mTNM systems, we performed a likelihood ratio χ^2^ test to evaluate homogeneity within the two TNM classifications [16]. The discriminatory ability and gradient monotonicity of the two classifications were estimated using a linear trend χ^2^ test [16]. Additionally, the discriminatory ability of the two TNM classifications was assessed using the Akaike information criteria (AIC) test (e.g., a smaller AIC score demonstrates a model that is more appropriate for evaluating prognosis) [16]. In all analyses, differences with *P* values < 0.05 were considered statistically significant.

## Results

### Comparison of characteristics of patients in the SEER and FMUUH databases

In the SEER database, 1833 patients met the screening criteria. Data from the FMUUH gastric cancer database were obtained using the same methods, which yielded 920 patients (Additional file 1: Figure S1). In all, the two databases included 2753 N3 patients. The median follow-up period for all patients was 19 months (range 1–304 months). The median follow-up periods in patients in the SEER and FMUUH databases were 16 months (range 1–304 months) and 25 months (range 1–177 months), respectively.

The relevant patient and tumor characteristics obtained from the two databases in addition to the combined dataset are presented in Table 1. Significant differences were identified in mean age, gender frequency, the proportions of each ethnicity, tumor histology, median tumor size, pT and overall staging, the type of gastrectomy performed and the median number of LNs retrieved. There was no significant difference in pT-stage-T2, N3 stage, pIIIa and pIIIb stage tumors or in the median number of MLNs. The median number of MLNs in all of the patients was 14 (range 7–90). There was no significant difference in the median number of MLNs between patients in the SEER and FMUUH databases (*P* = 0.469). The median number of LNs examined in all patients was 26 (range, 16–90). In patients in the SEER and FMUUH databases, the median number of LNs examined was 24 (range 16–90) and 30 (range 16–80), respectively (*P* < 0.001).

**Table 1:** Demographics of the patient population

Characteristic	SEER	FMUUH	*P* value	Overall
Mean age, y (%)	64.2 ± 13.7	58.9 ± 12.0	< 0.001	62.4 ± 13.4
Gender, *n* (%)				
Male	1024 (55.9)	674 (73.3)	< 0.001	1698 (61.7)
Female	809 (44.1)	246 (26.7)		1055 (38.3)
Ethnicity, *n* (%)				
White	662 (36.1)	0	< 0.001	662 (24.0)
Asian	555 (30.3)	920 (100)	< 0.001	1475 (53.6)
Black	251 (13.7)	0	< 0.001	251 (9.1)
Hispanic	346 (18.9)	0	< 0.001	346 (12.6)
Native American	19 (1.0)	0	< 0.001	19 (0.7)
Histology, *n* (%)				
G1/G2	263 (14.3)	184 (20.0)	< 0.001	447 (16.2)
G3/G4	1570 (85.7)	736 (80.0)		2306 (83.8)
Median tumor size, mm	60 (2–220)	70 (10–200)	0.005	60 (2–220)
T-category, *n* (%)				
T1	48 (2.6)	9 (1.0)	0.004	57 (2.1)
T2	83 (4.5)	32 (3.5)	0.194	115 (4.2)
T3	616 (33.6)	115 (12.5)	< 0.001	731 (26.6)
T4a	834 (45.5)	549 (59.7)	< 0.001	1383 (50.2)
T4b	252 (13.7)	215 (23.4)	< 0.001	467 (17.0)
N-category, *n* (%)				
N3a	1093 (59.6)	554 (60.2)	0.766	1647 (59.8)
N3b	740 (40.4)	366 (39.8)		1106 (40.2)
Stage, *n* (%)				
IIB	39 (2.1)	7 (0.8)	0.008	46 (1.7)
IIIA	59 (3.2)	25 (2.7)	0.471	84 (3.1)
IIIB	892 (48.7)	421 (45.8)	0.150	1313 (47.7)
IIIC	843 (46.0)	467 (50.8)	0.018	1310 (47.6)
Type of surgery, n (%)				
Proximal/distal gastrectomy	1274 (69.5)	350 (38.0)	< 0.001	1624
Total gastrectomy	559 (30.5)	570 (62.0)		1129
LNs examined, median	24 (16–90)	30 (16–80)	< 0.001	26 (16–90)
MLNs, median	14 (7–90)	14 (7–69)	0.469	14 (7–90)

*SEER*, Surveillance, Epidemiology, and End Results database; *FMUUH*, Fujian Medical University Union Hospital;*MLNs*, metastatic lymph nodes; *LNs*, lymph nodes

### Cut-off point survival analysis of N3a/N3b gastric cancer

**Fig. 1:**
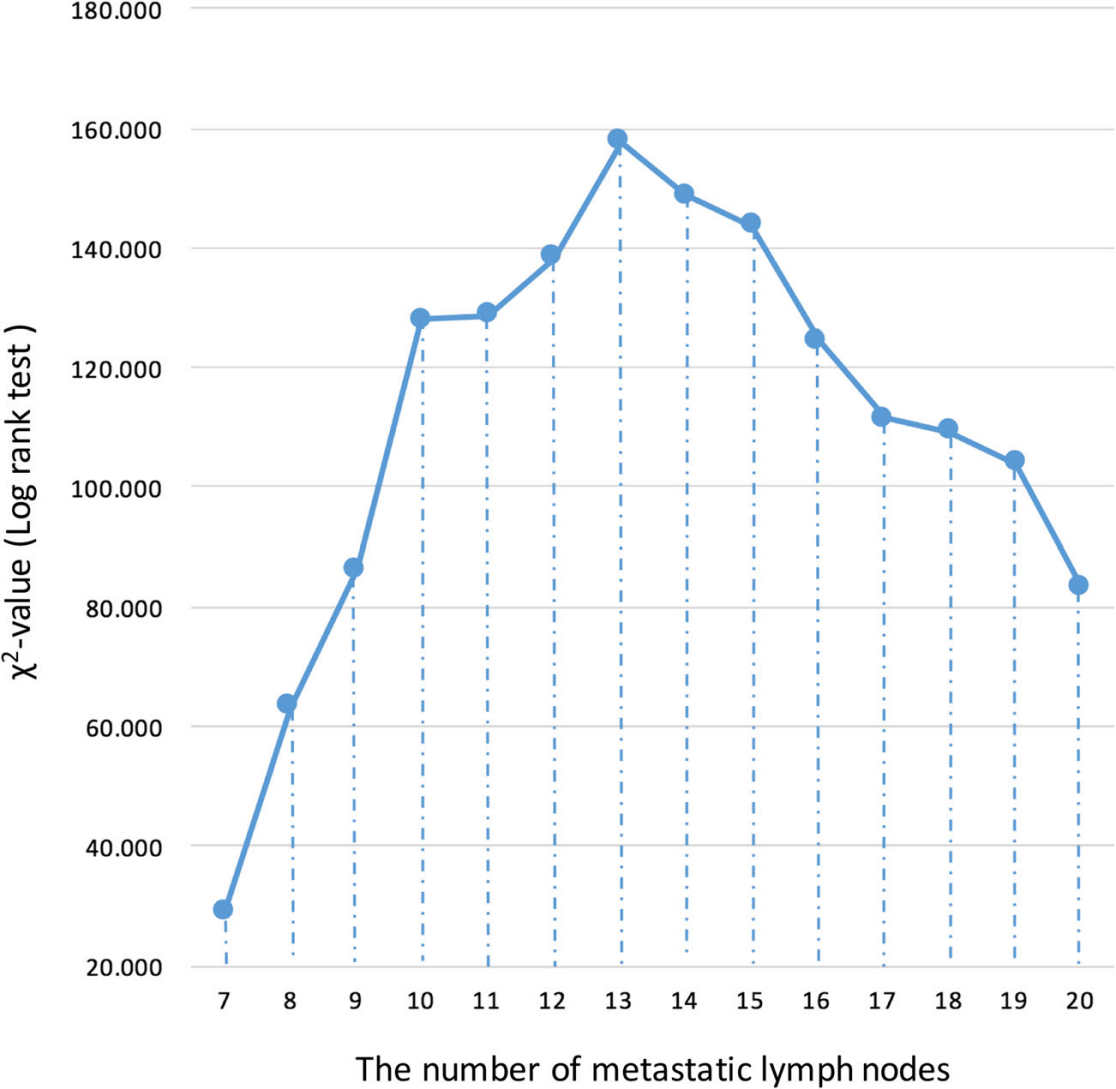
χ^2^ values in the cut-point survival analysis of patients with N3a/N3b gastric cancer

As shown in Fig. 1, we examined the ability of each cutoff value (according to the number of MLNs) to detect survival differences and found that a cutoff value of 13 MLNs most significantly stratified the prognoses of N3 patients into N3a and N3b groups (7–13 MLNs vs > 13 MLNs, five-year survival rate: 31% vs 15%; χ^2^ = 157.671, *P* = 3.65 × 10^− 36^) (Table 2). In addition, X-tile was used to verify that 13 MLNs was the optimal MLN count-based cutoff value to use in N3a/N3b gastric cancer (Additional file 2: Figure S2).

**Table 2:** Cut-point analysis to determine which cutoff value provided the most statistically significant difference in survival among patients with N3 gastric cancer

Cutoff value	Number of patients	5-year overall survival (%)	χ^2b^	*P* value^b^
≤ 7 vs > 7	215 vs 2538	35.8 vs 21.2	28.902	7.61 × 10^−8^
≤ 8 vs > 8	436 vs 2317	35.3 vs 19.9	63.197	1.87 × 10^−15^
≤ 9 vs > 9	646 vs 2107	34.0 vs 18.8	86.210	1.62 × 10^−20^
≤ 10 vs > 10	846 vs 1907	34.1 vs 17.1	127.873	1.20 × 10^−29^
≤ 11 vs > 11	1011 vs 1742	32.4 vs 16.5	128.609	8.26 × 10^−30^
≤ 12 vs > 12	1172 vs 1581	31.0 vs 16.0	138.384	6.01 × 10^−32^
≤ 13 vs > 13	1333 vs 1420	30.7 vs 14.5	157.671^a^	3.65 × 10^−36^
≤ 14 vs > 14	1501 vs 1252	29.2 vs 14.1	148.822	3.13 × 10^−34^
≤ 15 vs > 15	1647 vs 1106	28.3 vs 13.5	143.757	4.02 × 10^−33^
≤ 16 vs > 16	1797 vs 956	27.2 vs 13.2	124.495	6.57 × 10^−29^
≤ 17 vs > 17	1898 vs 855	26.3 vs 13.5	111.424	4.78 × 10^−26^
≤ 18 vs > 18	1996 vs 757	25.9 vs 12.8	109.305	1.39 × 10^−25^
≤ 19 vs > 19	2083 vs 670	25.6 vs 12.2	103.929	2.10 × 10^−24^
≤ 20 vs > 20	2165 vs 588	25.0 vs 12.5	83.056	7.97 × 10^−20^

^a^Corresponds to the cut point with the largest χ^2^ statistic

^b^Log rank test

### Overall survival in patients with 14–15 MLNs

**Fig. 2:**
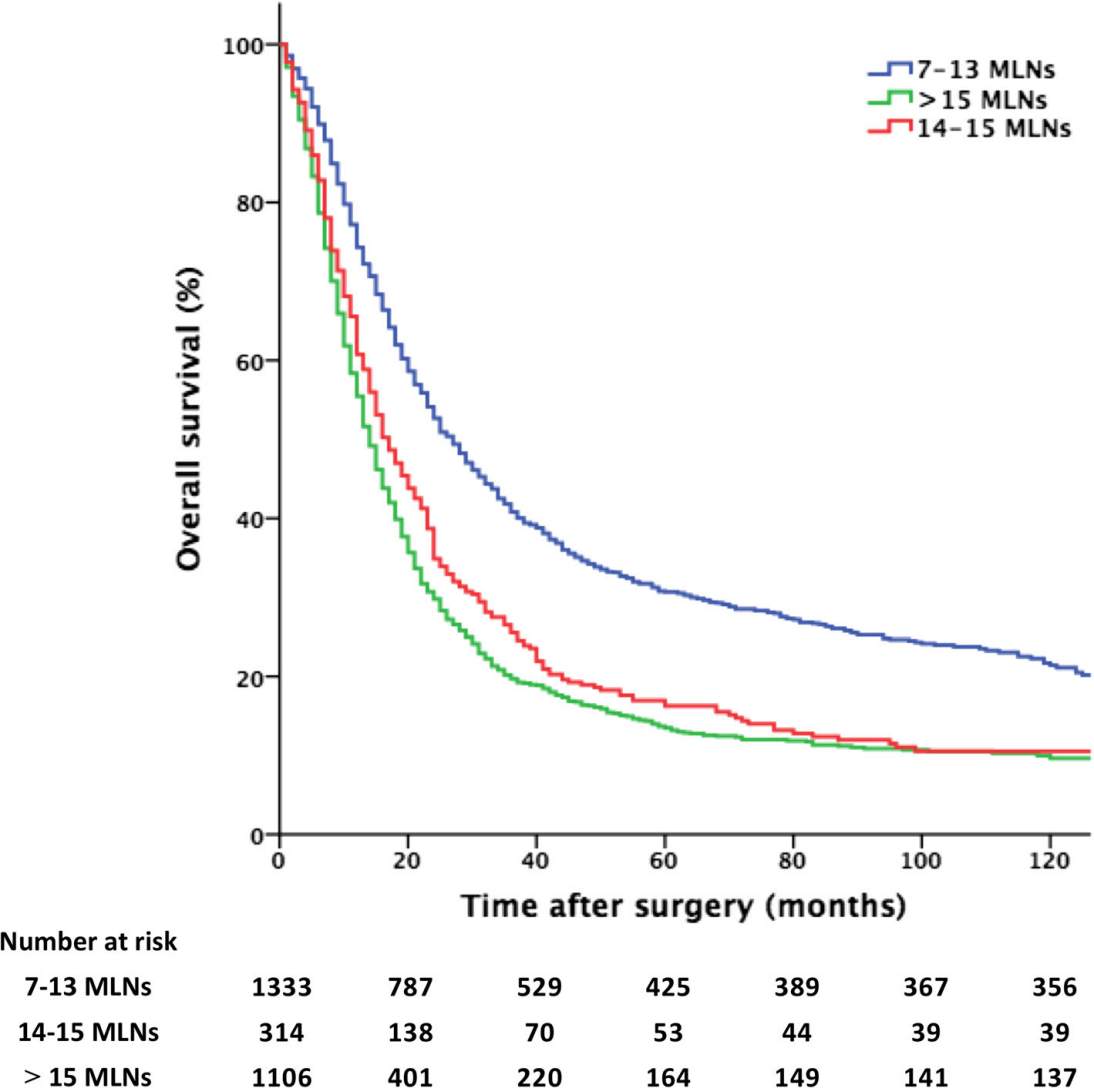
Overall survival curves in patients with 7–13, 14–15 and > 15 metastatic lymph nodes (MLNs)

Figure 2 shows the survival curves of patients with 7–13, 14–15 and > 15 MLNs. The five-year survival of patients with 14–15 MLNs was significantly worse than that observed in patients with 7–13 MLNs (16% vs 31%, respectively; *P* < 0.001) but similar to that observed in patients with > 15 MLNs (16% vs 14%, respectively; *P* = 0.078).

Based on these results, we proposed a modified N3 classification (mN3a: 7–13 MLNs, mN3b: > 13 MLNs) in which patients with 14–15 MLNs were incorporated into the mN3b group.

### Two-step multivariate analysis of overall survival in N3 patients

A univariate analysis and a two-step multivariate analysis of the included N3 patients were performed to further evaluate the validity of the mN3 classification (Table 3). In the univariate analysis, age, ethnicity, tumor size, pT classification, and the eighth N3 and mN3 classifications were significantly correlated with survival. In the first step of the multivariate analysis, age, ethnicity, tumor size, pT and the eighth N3 classification were demonstrated to be independent prognostic factors. However, when both the eighth N3 and the mN3 classifications were incorporated into the second step of the multivariate analysis, only the mN3 classification remained significant (HR 1.51, *P* < 0.001), while the eighth N3 classification disappeared (HR 1.13, *P* = 0.083).

**Table 3:** Univariate and multivariate analyses were performed using Cox’s proportional hazard model in patients with N3 gastric cancer

Characteristic	Univariate analysis			Multivariate analysis 1		Multivariate analysis 2	
	5-year OS (%)	χ2	P	HR (95% CI)	P	HR (95% CI)	P
Age at diagnosis, y		79.168	< 0.001				
< 65	27.9			1		1	
≥ 65	16.3			1.47 (1.35–1.60)	< 0.001	1.47 (1.35–1.60)	< 0.001
Gender		0.617	0.432				
Male	22.2						
Female	22.6						
Ethnicity		80.923	< 0.001				
White	14.4			1		1	
Asian	27.4			0.66 (0.59–0.73)	< 0.001	0.66 (0.60–0.73)	< 0.001
Black	15.8			1.02 (0.88–1.19)	0.781	1.03 (.88–1.20)	0.748
Hispanic	21.4			0.85 (0.74–0.98)	0.028	0.84 (0.72–0.97)	0.014
Native American	15.8			0.86 (0.52–1.42)	0.563	0.83 (0.50–1.36)	0.454
Histology		0.223	0.637				
G1/G2	22.7						
G3/G4	22.3						
Tumor size, mm		17.131	< 0.001				
≤ 60	24.6			1		1	
> 60	20.0			1.02 (0.94–1.12)	0.581	1.01 (0.93–1.10)	0.864
T-category		46.870	< 0.001				
T1	45.5			1		1	
T2	39.1			1.18 (0.79–1.75)	0.431	1.12 (0.75–1.67)	0.579
T3	24.4			1.66 (1.18–2.34)	0.004	1.60 (1.14–2.26)	0.007
T4a	20.3			1.86 (1.32–2.61)	< 0.001	1.77 (1.26–2.48)	0.001
T4b	18.0			2.18 (1.54–3.10)	< 0.001	2.06 (1.45–2.93)	< 0.001
Type of surgery		1.255	0.263				
Proximal/distal gastrectomy	22.8						
Total gastrectomy	21.7						
N-category		143.8	< 0.001				
N3a	28.3			1		1	
N3b	13.5			1.56 (1.43–1.70)	< 0.001	1.13 (0.99–1.29)	0.083
Modified N-category		157.67	< 0.001				
Modified N3a	30.7					1	
Modified N3b	14.5					1.51 (1.32–1.73)	< 0.001

### Comparisons between the eighth TNM and mTNM classification systems

**Fig. 3:**
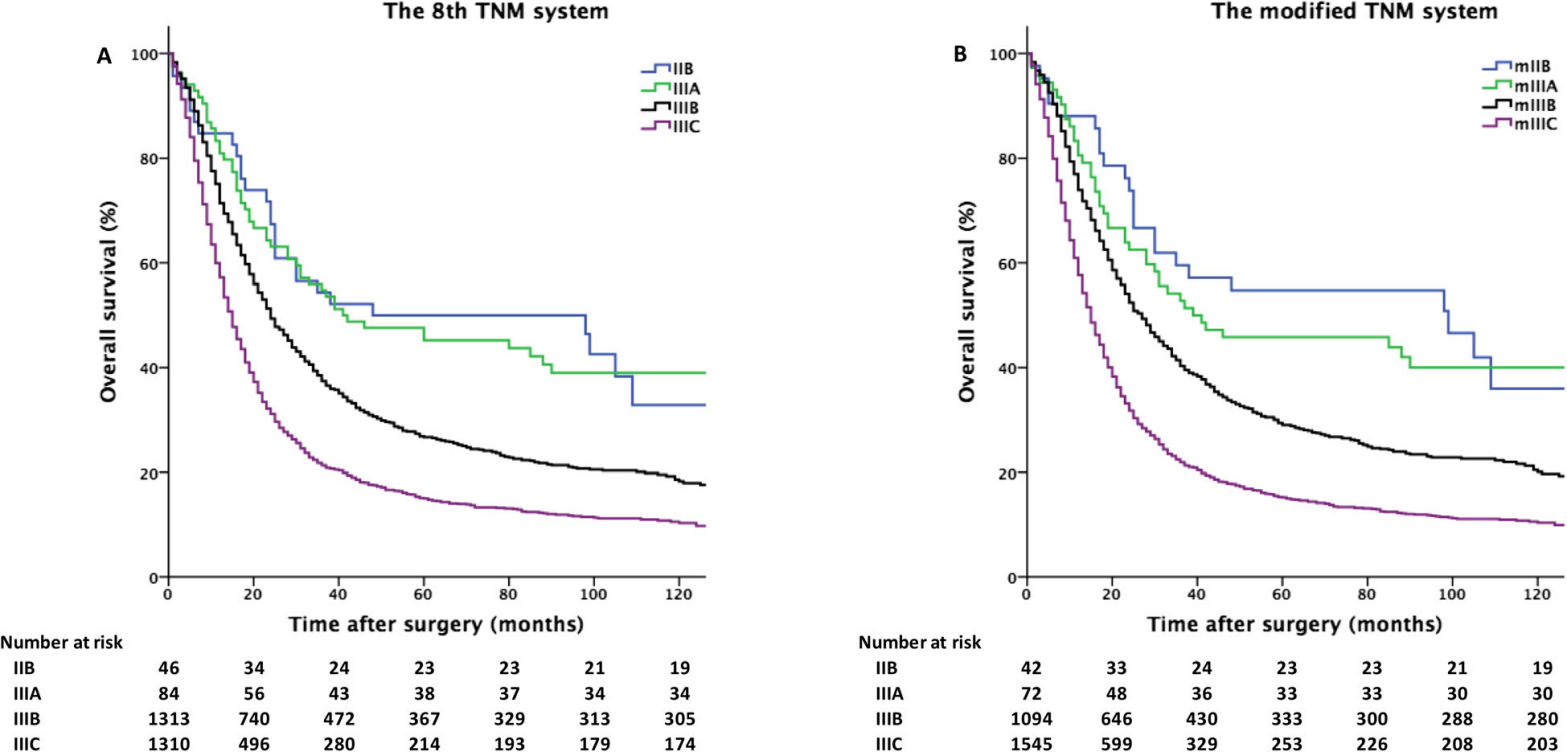
Overall survival curves for patients with N3 gastric cancer categorized according to **a** the 8th TNM and **b** modified TNM (mTNM) systems

Using mN3 staging, we modified the eighth TNM system. Based on the eighth TNM system, there were 46, 84, 1313 and 1310 patients with stage IIB, IIIA, IIIB and IIIC gastric cancer, respectively, and the five-year survival rates in these patients were 50, 45, 27 and 15%, respectively. Based on the mTNM classification, there were 42,72,1094 and 1545 patients with stage mIIB, mIIIA, mIIIB and mIIIC disease, respectively, and the five-year survival rates in these patients were 55, 46, 29 and 15%, respectively. Figure 3 shows the overall survival curves of the included patients with N3 gastric cancer categorized according to the eighth TNM and mTNM classifications.

The performance of the eighth TNM and the mTNM systems was evaluated using the linear trend χ^2^, likelihood ratio χ^2^, and the AIC tests, as presented in Table 4. Homogeneity was better in the mTNM system than in the eighth TNM system (likelihood ratio χ^2^ score, 102.796 vs 76.671) as were discriminatory ability and the monotonicity of the gradients (linear trend χ^2^ score, 97.225 vs 74.252). Furthermore, the mTNM classification had a smaller AIC score (32,195.19 vs 32,351.28), indicating optimum prognostic stratification (a smaller AIC score demonstrates a model that is more appropriate for evaluating prognosis).

**Table 4:** Comparison of the performance of the eighth edition TNM staging system and the modified TNM staging system

	Linear trend χ^2^ (*P* value)	Likelihood ratio χ^2^ (*P* value)	AIC
The eighth TNM system	76.671 (< 0.001)	74.252 (< 0.001)	32,351.28
The modified TNM system	102.796 (< 0.001)	97.225 (< 0.001)	32,195.19

*AIC* Akaike information criteria

## Discussion

Since the first TNM classification system for gastric cancer was applied in 1968 [17], the definition of N3 gastric cancer has been updated and revised several times. The third edition of the UICC/AJCC TNM system defined N3 as para-aortic or hepatoduodenal node metastasis [18]. The criterion was based on intraoperative clinical observations, which were associated with a high level of subjectivity; therefore, the fourth TNM classification dropped the N3 category and reclassified these patients as M1 [19]. Investigators later found that the number of MLNs is a good indicator of the extent of LN metastases [20, 21]. Thus, based on the number of MLNs, the fifth and sixth TNM classification defined N3 as > 15 MLNs [1, 22]. In addition to the UICC/AJCC TNM system, the JCGC classification is another internationally authoritative classification for gastric cancer. The 13th JCGC system divided regional LN stations into three tiers based on the location of the MLNs relative to the primary tumor and classified N3 as metastases in Group 3 LNs [3]. Numerous studies have confirmed that basing N classification on the number of MLNs is superior to using anatomical N staging in terms of objectivity, feasibility, reproducibility and prognostic accuracy [23, 24]. Hence, in the 14th JCGC, the anatomical-based N stage was altered to the numeric N stage used in the seventh UICC/AJCC classification [4]. In the seventh TNM system, N3 was defined as ≥7 MLNs and split into N3a and N3b based on the cutoff value used for N2/N3 in the fifth/sixth TNM system [5]. During the formulation of the seventh TNM classification, there were no convincing data showing that classifying cases as N3a and N3b had a significant impact on survival in Western patients. Accordingly, the N3 subgroup failed to be an individual determinant of the final TNM stage in this edition [8].

In all staging systems used in gastric cancer, N3 gastric cancer is recognized as an advanced gastric cancer with nodal metastases. Patients with N3 gastric cancer account for a large proportion of patients in China and the United States [25]. Having an accurate stratification for N3 patients would be conducive to making individualized treatment strategies in patients with different illness statuses and improve the prognoses of these patients. Although the seventh TNM classification is widely acknowledged and used, the validity of regarding N3 as a single category during stage grouping remains controversial [6, 26]. Many researchers have suggested that N3b tumors are associated with worse outcomes than N3a tumors and that a N3 subclassification should therefore be used for final staging [27, 28]. In addition, Komatsu et al. found that a positive LN ratio was useful for stratifying prognoses and evaluating the extent of local tumor clearance in patients with N3 gastric cancer [29]. However, one of the drawbacks of using the LN ratio is that there are no standardized categories for this metric in the literature, and this impedes the spread and application of the LN ratio. Considering usability and reproducibility, the eighth TNM system still uses a numeric N staging system [9]. Moreover, based on a survival analysis of 25,411 patients with gastric cancer, the International Gastric Cancer Association separated N3 into N3a and N3b in their final pathologic staging analysis [8]. Our data also indicate that N3a and N3b may represent diseases with differing severity, as the two groups show significant prognostic differences. However, the overall survival rates of N3a and N3b patients in the current study are distinct from that in previous investigations. This may be due to differences in ethnic composition, the period of patient enrollment or the treatment outcomes of the two datasets.

Currently, the presence of 15 MLNs is defined as the cutoff for N3a/N3b gastric cancer. This system was based on a retrospective study of 477 patients performed by the German Gastric Cancer Study Group [10]. This study, which was performed 20 years ago, has some limitations, including the fact that it was ethnically homogenous and contained a small sample of patients with > 7 MLNs (219 patients). It is therefore unclear whether a cutoff of 15 MLNs is appropriate for both Western and Eastern patients with N3 gastric cancer. Although many studies have examined the cutoff for LN staging in gastric cancer, few scholars have discussed the rationality of the existing N3a/N3b cutoff [30]. For the first time, we have specifically analyzed and determined the optimal subclassification of patients with N3a/N3b gastric cancer. We developed an international dataset consisting of 2753 Western and Eastern patients with N3 gastric cancer and used the log-rank test to demonstrate that 13 MLNs is the optimal MLN cutoff value for distinguishing N3a/N3b gastric cancer. Accordingly, we proposed a modified N3 classification system and performed a two-step multivariate analysis to verify that the prognostic significance of this mN3 classification was superior to that of the eighth N3 classification system. Importantly, we show that homogeneity, discriminatory ability, and the monotonicity of gradients is better in the mTNM system than the eighth edition system. We suggest that the superior results of prognostic assessments obtained using the mTNM system are attributable to its improved N3 staging accuracy.

An ideal staging system should also be universally applicable. Gastric cancers exhibit differences in biological behavior in addition to the extent of surgery required for treatment and the pathological diagnosis of examined LNs; therefore, there are remarkable differences in stage-specific outcomes between Eastern and Western patients [8, 31]. The success of the current study lies in the statistical power provided by the merger of large databases from two different countries. In this study, we found that there were significant differences between the SEER and FMUUH datasets in age, gender, ethnicity, histology, tumor size, T and overall staging, the type of surgery and the number of LNs examined. Thus, establishing an international database not only increases the numbers available for analysis but also significantly enhances the representativeness of the mN3 staging system proposed in the present study [32]. In addition, only patients in whom > 15 LNs were retrieved and complete five-year follow-up data were available were included, and this ensures the accuracy of the results obtained in this study.

Some shortcomings of this study should be noted. Because incomplete information was available regarding adjuvant therapy in the SEER database, the effects of adjuvant treatment were not evaluated. In addition, the study group cases were accessioned for 20 years, during which time diagnostic methods and treatment strategies have changed, and this also limits the strength of our observations.

## Conclusions

In summary, we first specifically analyzed and determined the MLN count cutoff value that most accurately distinguished N3a/N3b gastric cancer. To perform this analysis, we used a large sample of Eastern and Western patients, and we subsequently confirmed its prognostic significance. To obtain better prognostic stratification, we suggest adjusting the cutoff for subclassification of N3 gastric cancer to 13 MLNs.

## Additional files

Additional file 1: **Figure S1.** Flowchart illustrating the selection criteria used to identify the included patients. (JPG 2270 kb)

Additional file 2: **Figure S2.** X-tile analysis showing that the optimal cutoff value for subclassification of N3 gastric cancer is 13 metastatic lymph nodes (MLNs). (JPG 575 kb)

## Abbreviations

AIC: Akaike information criteria.
FMUUH: Fujian Medical University Union Hospital
JCGC: Japanese Classification of Gastric Cancer
LN: lymph node
MLN: metastatic lymph node
mN3, mTNM: modified N3, modified tumor-node-metastasis stage
SEER: the Surveillance, Epidemiology, and End Results database
UICC/AJCC: Union for International Cancer Control/American Joint Committee on Cancer

## References

[1] Sobin LH, Wittekind CUICCTNM (1997). Classification of malignant tumors, 5th edn. Berlin Heidelberg.

[2] Japanese Research Society for Gastric Cancer (1995). Japanese classifi- cation of gastric carcinoma. First English Ed.

[3] Japanese Gastric Cancer Association (1998). Japanese classification of gastric carcinoma: 2nd English edition. Gastric Cancer.

[4] Japanese Gastric Cancer Association (2011). Japanese classification of gastric carcinoma – 3rd English edition. Gastric Cancer.

[5] Edge SB, Byrd DR, Compton CC (2010). AJCC Cancer staging handbook.

[6] Ahn HS, Lee HJ, Hahn S, Kim WH, Lee KU, Sano T (2010). Evaluation of the seventh American joint committee on Cancer/International Union against Cancer classification of gastric adenocarcinoma in comparison with the sixth classification. Cancer.

[7] Jun KH, Lee JS, Kim JH, Kim JJ, Chin HM, Park SM (2014). The rationality of N3 classification in the 7th edition of the International Union against Cancer TNM staging system for gastric adenocarcinomas: a case-control study. Int J Surg.

[8] Sano T, Coit DG, Kim HH, Roviello F, Kassab P, Wittekind C (2017). Proposal of a new stage grouping of gastric cancer for TNM classification: international gastric Cancer association staging project. Gastric Cancer.

[9] Ajani JA, In H, Sano T, Amin MB (2016). Stomach. AJCC Cancer staging Manual.8th ed.

[10] Roder JD, Böttcher K, Busch R, Wittekind C, Hermanek P, Siewert JR (1998). Classification of regional lymph node metastasis from gastric carcinoma. German Gastric Cancer Study Group Cancer.

[11] Wittekind C (2015). The development of the TNM classification of gastric cancer. Pathol Int.

[12] health D, Men a (2001). Ageing and health.

[13] Smith DD, Schwarz RR, Schwarz RE (2005). Impact of total lymph node count on staging and survival after gastrectomy for gastric cancer: data form a large US-population database. J Clin Oncol.

[14] Camp RL, Dolled-Filhart M, Rimm DL (2004). X-tile: a new bio-informatics tool for biomarker assessment and outcome- based cut-point optimization. Clin Cancer Res.

[15] Sun Z, Wang ZN, Zhu Z, Xu YY, Xu Y, Huang BJ (2012). Evaluation of the seventh edition of American joint committee on Cancer TNM staging system for gastric cancer: results from a Chinese monoinstitutional study. Ann Surg Oncol.

[16] Yoon HM, Ryu KW, Nam BH, Cho SJ, Park SR, Lee JY (2012). Is the new seventh AJCC/UICC staging system appropriate for patients with gastric cancer?. J Am Coll Surg.

[17] International Union Against Cancer (UICC) (1968). TNM classification of malignant tumors. Geneva: UICC.

[18] Harmer MH, International Union Against Cancer (UICC) (1982). TNM classification of malignant tumors.

[19] International Union Against Cancer (UICC). In: Hermanek P, Sobin LH, eds. TNM classification of malignant tumors, 4th edn. Berlin, New York: springer, 1987; revised 1992.

[20] Ichikura T, Tomimatsu S, Okusa Y, Uefuji K, Tamakuma S (1993). Comparison of the prognostic significance between the number of metastatic lymph nodes and nodal stage based on their location in patients with gastric cancer. J Clin Oncol.

[21] Adachi Y, Kamakura T, Mori M, Baba H, Maehara Y, Sugimachi K (1994). Prognostic significance of the number of positive lymph nodes in gastric carcinoma. Br J Surg.

[22] Sobin LH, Wittekind C (2002). UICC TNM classification of malignant tumors.

[23] Ichikura T, Tomimatsu S, Uefuji K, Kimura M, Uchida T, Morita D (1999). Evaluation of the new American joint committee on Cancer/international union against cancer classification of lymph node metastasis from gastric carcinoma in comparison with the Japanese classification. Cancer.

[24] Celen O, Yildirim E, Gulben K, Berberoglu U (2003). Prediction of survival in gastric carcinoma related to lymph node grading by the new American joint committee on Cancer/union international Contre le Cancer system or the Japanese system. Eur J Surg Suppl.

[25] Li P, Huang CM, Zheng CH, Russo A, Kasbekar P, Brennan MF (2018). Comparison of gastric cancer survival after R0 resection in the US and China. J Surg Oncol.

[26] Marrelli D, Morgagni P, de Manzoni G, Coniglio A, Marchet A, Saragoni L (2012). Prognostic value of the 7th AJCC/UICC TNM Classi cation of noncardia gastric Cancer analysis of a large series from specialized Western centers. Ann Surg.

[27] Fang WL, Huang KH, Chen JH, Lo SS, Hsieh MC, Shen KH (2011). Comparison of the survival difference between AJCC 6th and 7th editions for gastric Cancer patients. World J Surg.

[28] Yeh CN, Wang SY, Hsu JT, Chiang KC, Cheng CT, Tsai CY (2015). N3 subclassification incorporated into the final pathologic staging of gastric cancer: a modified system based on current AJCC staging. Medicine (Baltimore).

[29] Komatsu S, Ichikawa D, Miyamae M, Kosuga T, Okamoto K, Arita T (2016). Positive lymph node ratio as an Indicator of prognosis and local tumor clearance in N3 gastric Cancer. J Gastrointest Surg.

[30] Zhang J, Jiang K, Liu Y, Ye Y, Lv L, Shen Z (2014). Proposal of a new lymph node staging system for gastric cancer: study from two institutions in China. Med Oncol.

[31] Qiu MZ, Wang ZQ, Zhang DS, Liu Q, Luo HY, Zhou ZW (2011). Comparison of 6th and 7th AJCC TNM staging classification for carcinoma of the stomach in China. Ann Surg Oncol.

[32] Woo Y, Goldner B, Ituarte P, Lee B, Melstrom L, Son T (2017). Lymphadenectomy with optimum of 29 lymph nodes retrieved associated with improved survival in advanced gastric Cancer: a 25,000-patient international database study. J Am Coll Surg.

