# Supplementary figures and images for: A modified subclassification to evaluate the survival of patients with N3 gastric cancer: an international database study
Source: BMC Cancer. 2019 Jan 7;19:21. doi: 10.1186/s12885-018-5187-7 (PMC6323664; doi:10.1186/s12885-018-5187-7)

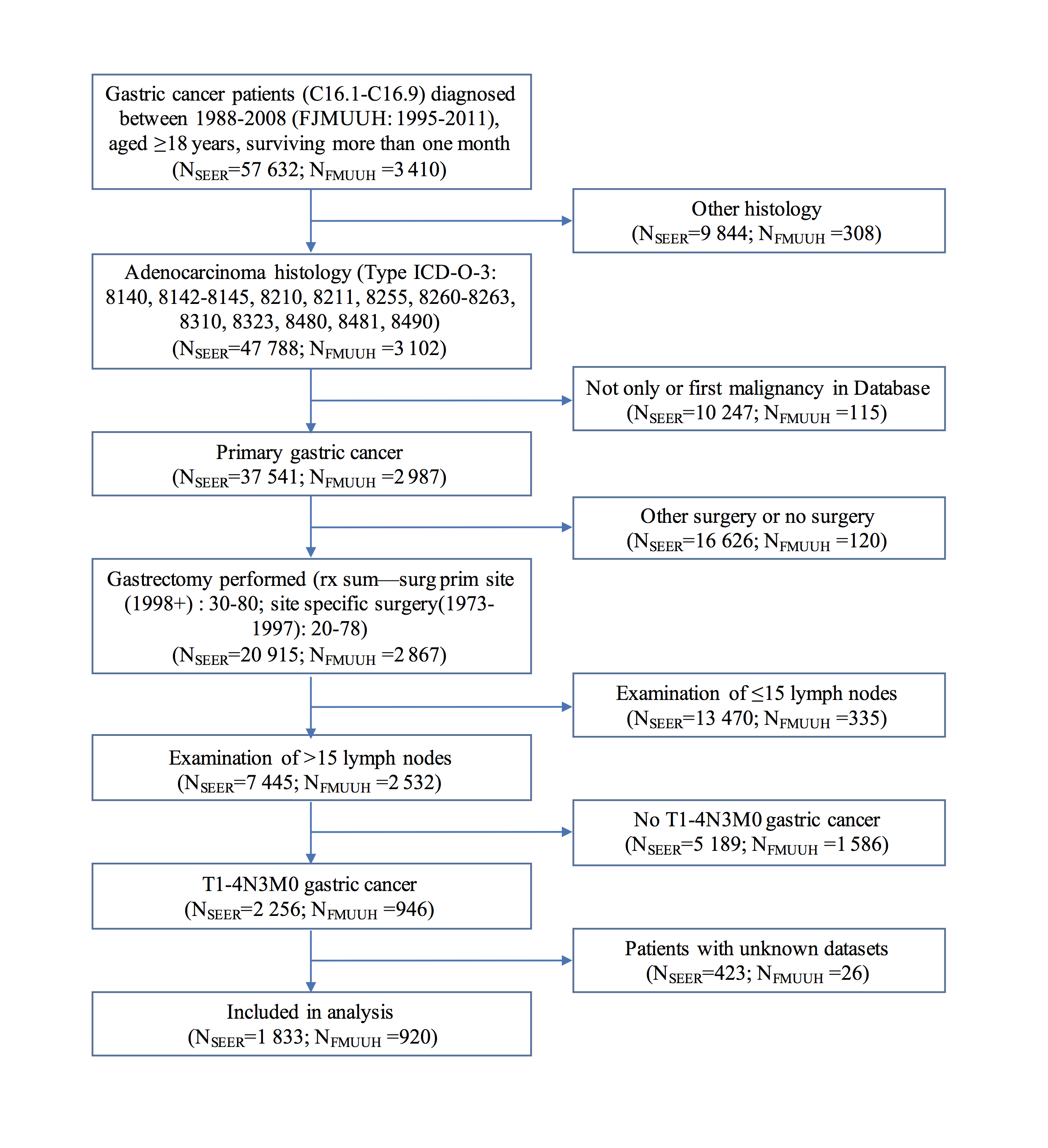

Supplement: Supplementary file 1 — Figure S1. Flowchart illustrating the selection criteria used to identify the included patients. (JPG 2270 kb) [file 12885_2018_5187_MOESM1_ESM.jpg]

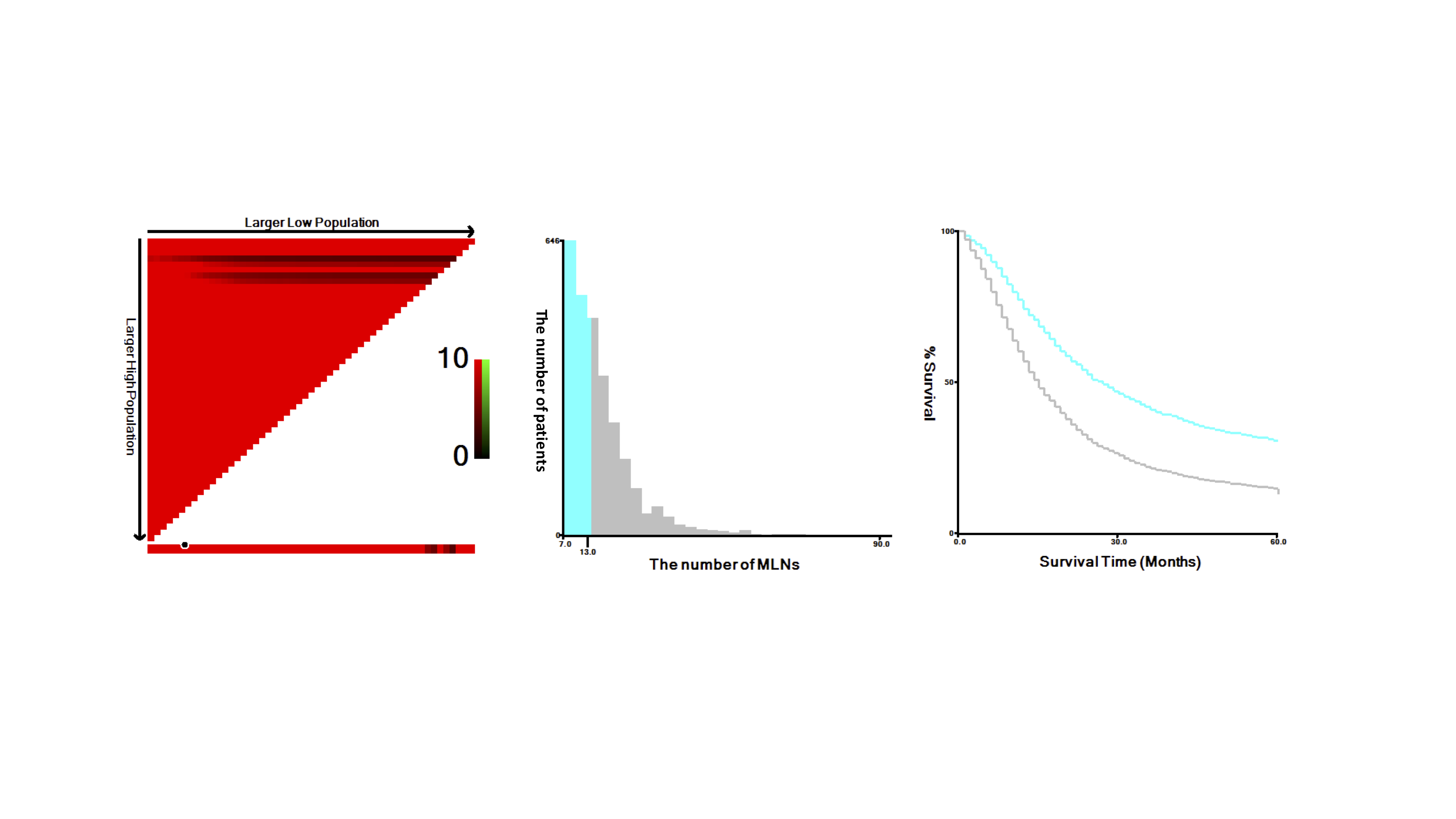

Supplement: Supplementary file 2 — Figure S2. X-tile analysis showing that the optimal cutoff value for subclassification of N3 gastric cancer is 13 metastatic lymph nodes (MLNs). (JPG 575 kb) [file 12885_2018_5187_MOESM2_ESM.jpg]
